# Development of a supportive-educative nursing model based on health promotion for independent wound care in diabetic foot ulcer patients: A cross-sectional study

**DOI:** 10.1016/j.ijnsa.2026.100504

**Published:** 2026-02-09

**Authors:** Novita Verayanti Manalu, Esti Yunitasari, Sriyono Sriyono, Nursalam Nursalam, Ferry Efendi, Yunus Elon

**Affiliations:** a Faculty of Nursing, Universitas Airlangga, Surabaya, Indonesia; b Faculty of Nursing, Universitas Advent Indonesia, Bandung, Indonesia

**Keywords:** Diabetic foot ulcers, Self-care, Wound care, Nursing models, Health promotion, Community health nursing

## Abstract

**Background:**

Diabetic foot ulcers are a chronic complications of diabetes associated with substantial morbidity, amputation risk, and reduced quality of life. Promoting the patients’ capacity for independent wound care is a key component of long-term management. Although health promotion and supportive–educative nursing approaches are widely advocated in nursing, empirically informed models specifically addressing independent wound care in diabetic foot ulcer patients remain limited, particularly in low-income and middle-income countries.

**Objective:**

To develop a supportive–educative nursing model based on health promotion for independent wound care in patients with diabetic foot ulcers.

**Design:**

A cross-sectional study was conducted during the model development phases.

**Settings:**

Twenty-two community health centers in Bandar Lampung, Indonesia.

**Participants:**

A total of 130 patients with grade 1–2 diabetic foot ulcers were recruited using purposive sampling, based on predefined inclusion and exclusion criteria.

**Methods:**

This study represents the development phase of this model. Quantitative data were collected using validated questionnaires assessing individual factors, support and facilities factors, nursing factors, supportive–educative nursing, patient commitment, and independent wound care behaviors. Structural equation modeling using partial least squares was applied to examine associations among constructs and inform model development.

**Results:**

Individual (β = 0.399, *p* = 0.002), support and facilities (β = 0.227, *p* = 0.022), and nurse (β = 0.296, *p* < 0.001) factors were significantly associated with supportive–educative nursing. Supportive–educative nursing was strongly associated with patient commitment (β = 0.724, *p* < 0.001), which in turn was associated with independent wound care behaviors (β = 0.486, *p* < 0.001). Direct associations between support and facilities and nurse factors and independent wound care were not significant, consistent with an indirect pathway through supportive–educative nursing and patient commitment.

**Conclusions:**

This study proposes a health promotion–based supportive–educative nursing model in which patient commitment functions as a key mediating pathway linking nursing support to independent wound care. These findings provide an empirically informed framework to guide nursing practice and support future longitudinal and interventional research.

**Registration:**

The Health Research Ethics Committee of Universitas Airlangga (Ref. No. 3664-KEPK), Investment and One-Stop Integrated Services Agency (Reference number 1871/070/06354/SKP/III.16/II/2025), and Bandar Lampung City Health Agency (Reference number B/400.7.22/III.02.V/02/2025).

**What is already known**

• Diabetic foot ulcers require long–term self–care management; however, many patients remain dependent on nurses for wound care.
• Supportive–educative nursing, based on Orem’s theory, promotes patient independence but is rarely integrated with health promotion approaches.
• Evidence remains limited on how patient, nurse, and environmental factors are jointly related to self–care independence in diabetic foot ulcer management, particularly in community settings.

Alt-text: Unlabelled box dummy alt text

**What this paper adds**

• This study developed a supportive–educative nursing model grounded in health promotion for independent wound care in patients with diabetic foot ulcers.
• The model identified patient commitment as a key mediating pathway linking individual, support and facilities, and nursing factors with independent wound care behaviors.
• The findings offer a theoretically grounded and transferable framework to inform nurse-led empowerment strategies in community and primary care settings.

Alt-text: Unlabelled box dummy alt text

## 1 Introduction

Diabetic foot ulcers remain a common and serious complication of diabetes mellitus and are associated with prolonged wound healing, increased risk of infection and amputation, and substantial reductions in quality of life (Yammine and Assi, 2019). Recent global estimates indicate a prevalence of approximately 6–10 %, with a disproportionate burden in low- and middle-income countries, where access to continuous wound care and preventive services is often limited (International Diabetes Federation, 2025). Beyond clinical treatment, growing evidence indicates that long-term Diabetic Foot Ulcer outcomes depend heavily on patients’ ability to consistently perform appropriate wound care behaviors consistently in daily life (Bus et al., 2020; Zhuang et al., 2024). However, many patients experience difficulties in maintaining independent wound because of limited knowledge, low confidence, insufficient motivation, and inadequate professional or family support (Joeliantina et al., 2024; Manalu et al., 2024).

The nursing and self-management literature emphasizes the importance of supportive–educative nursing in strengthening patients’ self-care capacity through structured education, guidance, and motivational support (Dehghan Nayeri et al., 2020; Osuchukwu et al., 2021). In this study, supportive–educative nursing is defined as nursing practices that systematically support patients’ learning, skill development, and behavioral change to enhance self-care agency and independence, consistent with Orem’s Self-Care Deficit Nursing Theory. Evidence from chronic disease management demonstrates that effective supportive–educative nursing is associated with improved self-care behaviors, reduced caregiver burden, and better health outcomes (Kusumaningrum et al., 2024). Within this framework, nurses function not only as care providers but also as facilitators who enable patients to acquire the knowledge, skills, and confidence required for long-term self-management.

Health promotion perspectives further extend this view by emphasizing empowerment, active participation, and sustained behavioral engagement as essential mechanisms underlying successful self-management of chronic conditions, including Diabetic Foot Ulcer care (Vakilian et al., 2021; Kaewjaladvilai, Chaiviboontham and Sumdaengrit, 2025). From a health promotion standpoint, patient commitment represents a central construct linking motivation and intention to sustained health behaviour. In this study, patient commitment refers to a patient’s sense of responsibility and action planning toward maintaining consistent wound care behaviors in daily life (Barenfeld et al., 2017).

Accumulating empirical evidence has indicated that independent wound care is influenced by multiple interacting determinants. These include individual factors, such as motivation, self-efficacy, and prior wound care experience; support and facilities factors, such as access to health services, availability of facilities, and social support; and nursing factors, including the supporting, teaching, guidance, and social support (Joeliantina et al., 2024; Manalu et al., 2024). However, these determinants have been frequently examined in isolation. There remains limited empirical work integrating individual, contextual, and nursing-related factors into a coherent nursing model that explains how they jointly contribute to independent wound care, particularly in community health settings and in low- and middle-income countries (Alaydrus, Suryawati and Harto, 2025).

Within this context, supportive–educative nursing and health promotion are viewed as complementary approaches that emphasize patient empowerment and active engagement in self-care. Rather than treating patient-centered care as a separate construct, this study conceptualizes patient-centered principles as operationalized through supportive–educative nursing practices and strengthened through patient commitment. Addressing the absence of an empirically informed integrative framework, this study aimed to examine the relationships among individual factors, support and facilities factors, nurse factors, supportive–educative nursing, patient commitment, and independent wound care behaviors to inform the development of a supportive–educative nursing model for Diabetic Foot Ulcer care.

### 1.1 The development of the supportive–educative nursing model

**Fig. 1:**
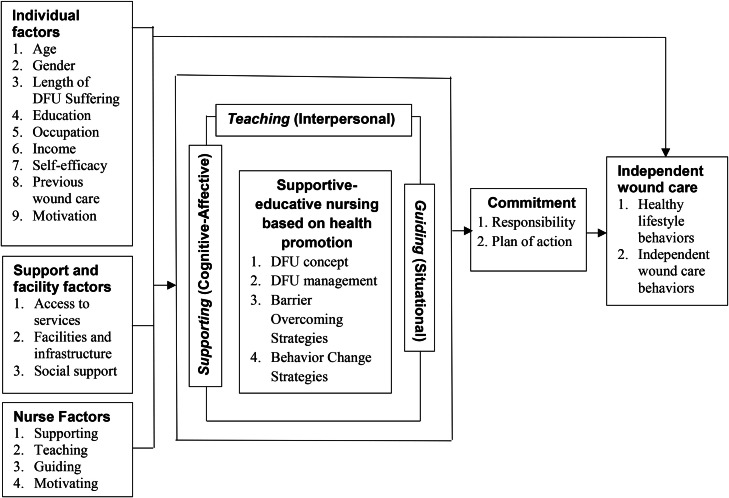
Conceptual Framework of Developing a Supportive-Educative Model Based on the Health Promotion for Independent Wound Care in Diabetic Foot Ulcer Patients.

Based on Orem’s Self-Care Deficit Nursing Theory and Pender’s Health Promotion Model, a conceptual framework was developed to illustrate the proposed relationships among individual factors, support and facilities factors, nurse factors, supportive–educative nursing, patient commitment, and independent wound care behaviors (Fig. 1). The framework integrates nursing and behavioral perspectives to support sustained self-care among patients with diabetic foot ulcers.

The model proposes that individual, contextual, and nurse-related factors influence supportive–educative nursing, which functions as the central nursing process that facilitates patient learning, skill development, and behavioral change. Consistent with the contemporary nursing literature, nurses are conceptualized as facilitators who enhance patients’ self-care capacity through education, guidance, and motivational support rather than acting solely as direct care providers (Kusumaningrum, Niken and Paramitta, 2024). Within this process, patient commitment is positioned as a key behavioral mediator through which supportive–educative nursing contributes to sustained independent wound care behaviors, reflecting the emphasis on empowerment, active participation, and intention in health promotion research (Zhuang et al., 2024; Nitichai, Harnirattisai and Hain, 2025).

The development of this model was informed by recent empirical evidence demonstrating that self-care in diabetic foot ulcer management is shaped by multiple interacting personal, contextual, and professional factors. Prior studies have indicated that these factors collectively influence patients’ engagement in foot self-care behaviors, adherence to recommended care, and overall capacity to manage chronic wounds (Joeliantina et al., 2024). Accordingly, the proposed model comprises six interrelated constructs: individual factors, support and facilities factors, nurse factors, supportive–educative nursing, patient commitment, and independent wound care behaviors.

### 1.2 Study hypotheses

Based on the conceptual framework described in Fig. 1, the following hypothesis was formulated:

H1 Individual, support and facilities, and nurse factors are positively associated with supportive–educative nursing.

H2 Supportive–educative nursing is positively associated with patient commitment.

H3 Patient commitment is positively associated with independent wound care behaviors.

H4 Supportive–educative nursing and patient commitment mediate the relationships between individual factors, support and facilities factors, nurse factors, and independent wound care behaviors.

## 2 Methods

### 2.1 Study design

This cross-sectional study was conducted as a model development study aimed at designing a supportive–educative nursing model for independent wound care in patients with diabetic foot ulcers. Guided by Orem’s Self-Care Deficit Nursing Theory and Pender’s Health Promotion Model and supported by current empirical evidence, the study was implemented in three sequential and interrelated steps.

First, key constructs relevant to independent wound care, including individual factors, support and facilities factors, nurse factors, supportive–educative nursing, patient commitment, and independent wound care behaviors, were identified and integrated into the model.

Second, measurement instruments were developed and refined to operationalize each construct, involving item generation based on theory and literature, expert review to establish content validity, and pilot testing to assess clarity, reliability, and preliminary construct validity.

Third, the proposed model was empirically tested and finalized using partial least squares structural equation Modeling to examine the relationships among constructs, with the results of both refinement of the supportive–educative nursing model and development of a related educational module to support patient self-care in diabetic foot ulcer management.

### 2.2 Participants and setting

This study was conducted in 22 community health centers (primary care facilities) in Bandar Lampung, Indonesia. These centers represent the first level of care within the national primary healthcare system and provide diabetes management and wound care services as part of the chronic disease program of the Ministry of Health.

Sample size estimation followed established guidelines for variance-based structural equation modeling, recommending a minimum of 5–10 participants per estimated parameter (Hair et al., 2022). Based on the 24 observed parameters, a minimum sample size of 120 participants is required. In total, 130 participants were recruited to account for potentially incomplete data.

Participants were selected using purposive sampling from patients attending the participating community health centers. The inclusion criteria were as follow: (1) diagnosis of grade 1 to early grade 2 diabetic foot ulcers; (2) random blood sugar >120 mg/dL, indicating impaired glycemic control relevant to wound healing; (3) age ≥18 years; (4) ability to read and write; and (5) willingness to participate. Random blood sugar was used because HbA1c testing is not routinely available in all community health centers, and the selected cut-off reflects common screening thresholds applied in primary care settings. Exclusion criteria included communication barriers or psychosocial disorders, severe clinical conditions requiring referral to advanced care, and a diagnosis of peripheral artery disease. The exclusion of peripheral artery disease aimed to ensure a more homogeneous sample with respect to wound aetiology and to reduce confounding factors related to ischaemic ulcers. The participants had no prior therapeutic relationship with the research team or were newly registered patients, thus minimizing potential response bias.

### 2.3 Measurements

All study constructs were measured using a structured self-administered questionnaire developed by the authors, adapted from validated instruments and grounded in the relevant theoretical frameworks and literature. The adaptation process involved item selection and modification, and items relevant to the local context were translated into Indonesian and adapted culturally. Ambiguous or redundant items were revised based on the expert feedback. Some of items were rated on a five-point Likert scale ranging from 1 (strongly disagree/never) to 5 (strongly agree/always), five-point ordinal scale (1=poor to 5=excellent), and a two-point binary scale (1=Yes and 0=No), with higher scores indicating higher levels of the measured construct. Detailed information regarding the source instruments, item composition, and Nadaptation process for each construct are provided in Supplementary Table S1.

#### 2.3.1 Six constructs of the questionnaire

Six questionnaire constructs were used to assess the determinants, nursing processes, and outcomes of independent wound care in patients with diabetic foot ulcers. All the constructs were measured using adapted, developed, and validated questionnaires. Details of the sources and adaptations of all instruments are provided in Supplementary Table S1.

Individual Factors (X1) assessed patients’ self-efficacy, motivation, and prior wound care experiences related to self-management behaviors. The construct consisted of 57 items, with higher scores indicating greater individual capacity for independent wound care (Bakken et al., 2000; Jiang et al., 2015; Chin et al., 2019; Wahengbam, 2019)

Support and facilities Factors (X2) measured contextual resources, including access to healthcare services, availability of facilities, and social support. This construct included 38 items, with higher scores reflecting stronger perceived environmental and social support (La Greca and Bearman, 2002; Hoseini-Esfidarjani et al., 2021).

Nurse Factors (X3) assessed patients’ perceptions of nursing education, guidance, emotional support, and motivation. The construct comprised 36 items, with higher scores indicating more effective nursing support (Hidalgo-Ruiz et al., 2023).

Supportive–Educative Nursing (X4) reflects nursing practices that support patient learning, skill development, and behavioral change, grounded in Orem’s Self-Care Deficit Nursing Theory. This construct consists of 61 items, with higher scores indicating stronger supportive–educative nursing practice (Polonsky et al., 1995; Glasgow et al., 2005; Huijg et al., 2014; Royal and Royal, 2017).

Patient Commitment (Y1) measures patients’ responsibility and action planning for consistent wound care and comprises 10 items. Higher scores indicated a stronger commitment to self-managed wound care (Schwarzer, 2016; Mahmoodi et al., 2021).

Independent Wound Care Behaviors (Y2) capture the patients’ ability to independently perform the recommended wound care activities. This outcome construct consisted of 26 items, with higher scores indicating greater wound care independence (Lecker et al., 2022; Ruiz-Mun ~oz et al., 2024; Setyawati et al., 2024).

#### 2.3.2 Validity and reliability

Content validity was established through expert review by nursing and wound care specialists who evaluated item relevance and clarity. Revisions were made based on expert feedback to ensure alignment the theoretical constructs.

To assess item clarity and internal consistency, a pilot test was conducted with participants who were not included in the final analysis to assess item clarity and internal consistency (Boateng and Catanzano, 2015). Internal consistency reliability for all constructs was acceptable, with Cronbach’s alpha values ranging from 0.747 to 0.957, indicating acceptable to excellent reliability (Hair et al., 2022).

Construct validity was further evaluated by assessing factor loadings, composite reliability, average variance extracted, and discriminant validity. These procedures are described in detail in the Statistical Analysis section.

### 2.4 Data collection

Data were collected between March and May 2025 at 22 community health centers in Bandar Lampung, Indonesia. The data collection process comprised participant recruitment, questionnaire administration, and model refinement. A total of 130 patients with diabetic foot ulcers who met the inclusion criteria were recruited from diabetes clinics and wound care units at the participating centers. Written informed consent was obtained from all participants after they received an explanation of the study. The participants then completed the validated, self-administered questionnaires under researcher supervision.

Following the survey, focus group discussions with eight patients and eight caregivers were conducted solely to refine the model; followed by expert consultation with wound care and diabetes specialists to ensure its clinical relevance and contextual applicability. This discussion is not a formal qualitative data analysis; no further data collection was conducted as this study used a cross-sectional design. Participants with incomplete responses were excluded from analysis. No imputation was performed because the missing data were minimal (<5 %) and were randomly distributed.

### 2.5 Statistical analysis

Data analysis was conducted using Partial Least Squares Structural Equation Modeling, which was selected because it is suitable for theory-informed model development and predictive analysis involving complex models with multiple latent constructs. This approach is particularly appropriate for exploratory research with relatively modest sample sizes and minimal distributional assumptions, making it more suitable than covariance-based structural equation modeling in the present research context.

The analysis followed a two-stage approach:

#### 2.5.1 Evaluation of the measurement model

The measurement model was used to establish the reliability and validity of the constructs. Indicator reliability was evaluated using factor loadings, with values ≥0.70 considered acceptable. Internal consistency reliability was assessed using both Cronbach’s alpha and composite reliability (CR), with values ≥0.70 indicating satisfactory reliability. Convergent validity was examined through average variance extracted, with values ≥0.50 considered adequate, while discriminant validity was evaluated using the Fornell-Larcker criterion to ensure that each construct was distinct from the others.

#### 2.5.2 Evaluation of the structural model

The structural model was evaluated by examining path coefficients, coefficients of determination (R²), and predictive relevance (Q²), while collinearity among constructs was assessed prior to evaluating the structural relationships. Hypotheses were tested using bootstrapping with 5000 resamples to assess the significance of both the direct and indirect effects.

#### 2.5.3 Hypothesis testing

Hypotheses were tested using a bootstrapping procedure to estimate the significance of direct and indirect (mediated) relationships among the constructs. Path coefficients and the corresponding confidence intervals were used to assess the strength and significance of the hypothesized associations. The mediation effects of supportive–educative nursing and patient commitment were examined in accordance with the proposed conceptual framework. All analyses were conducted at a significance level of *P* < 0.05.

### 2.6 Ethics

All study participants provided informed consent, and the study was carried out in compliance with the established ethical guidelines. The participants were thoroughly apprised of the study objectives, methodologies, and possible advantages. They received equitable and respectful treatment throughout the research procedure and their identities were safeguarded by omitting their names from the questionnaires during data collection. The Health Research Ethics Committee of Universitas Airlangga (reference number 3664-KEPK), The Investment and One-Stop Integrated Services Agency (reference number 1871/070/06354/SKP/III.16/II/2025), and Bandar Lampung City Health Agency (reference number B/400.7.22/III.02.V/02/2025) approved this study. To maintain confidentiality, the completed surveys were securely held in a locked folder overseen by the researcher.

## 3 Results

### 3.1 Sample characteristics

The results of the descriptive analysis (Table 1) demonstrated that the majority of diabetic foot ulcer patients were aged 55–65 years (36.3 %), followed by those aged 46–55 years (30 %), who fell into the pre-elderly and early elderly categories. Furthermore, the majority of patients were male (62.3 %), and the majority of the sample had diabetic foot wounds lasting >4 weeks (72.9 %). The predominant education level of patients was elementary school to junior high school (52.3 %), which is compulsory in Indonesia. Most occupations were housewives/unemployed (57.5 %). Many patients were unemployed due to their advanced age (38.5 %), but a significant number worked in the private sector (33.8 %), with the average income of most patients below the minimum wage (46.2 %).

**Table 1:** Frequency and Percentage of Participants' Characteristics (*n* = 130).

Characteristics	Frequency (f)	Percentage (%)
Age (years)		
18 – 25	0	0
26 – 35	2	1,5 %
36 – 45	5	3,8 %
46 – 55	39	30 %
56 – 65	47	36,2 %
> 65	37	28,5 %
Gender		
Male	81	62,3 %
Female	49	37,7 %
Long-time suffering from DFU		
< 4 weeks	27	20,8 %
> 4 weeks	103	79,2 %
Education		
Elementary – Middle School	68	52,3 %
High School	49	37,7 %
College	13	10 %
Occupation		
Civil servant	8	6,2 %
Private employee	44	33,8 %
Housewife	28	21,5 %
Unemployed	50	38,5 %
Income (> US $163 <)		
< 2,7 Million Rupiah/month	60	46,2 %
2,7 Million Rupiah/month	6	4,5 %
> 2,7 Million Rupiah/month	21	16,2 %
No income	43	33,1 %

*DFU: Diabetic Foot Ulcer.

### 3.2 Result of convergent validity testing

Convergent validity was assessed to determine how well each indicator captured its underlying latent constructs. All indicators demonstrated strong factor loadings, ranging from 0.705 to 0.969, exceeding both the minimum criterion of 0.50 and the recommended threshold of 0.70, indicating that each item adequately represented its respective construct. Similarly, the Average Variance values for all constructs ranged from 0.628 to 0.931, surpassing the minimum requirement of 0.50 (Hair et al., 2022), confirming satisfactory convergent validity (Table 2).

**Table 2:** Results of convergent validity testing (*n* = 130).

Latent Variabel		Observational Variables	Loading Factor	Average Variance Extracted
Individual Factors (X1)	X1.7	Self-efficacy	0.845	0.729
	X1.8	Previous wound care	0.880	
	X1.9	Motivation	0.773	
Support and Facilities Factors (X2)	X2.1	Access to services	0.813	0.628
	X2.2	Facilities	0.764	
	X2.3	Social support	0.800	
Nursing Factors (X3)	X3.1	Nurse support	0.867	0.700
	X3.2	Teaching	0.847	
	X3.3	Guidance	0.705	
	X3.4	Nurse motivation	0.913	
Supportive-Educative Nursing (X4)	X4.1	DFU concept	0.724	0.775
	X4.2	DFU management	0.909	
	X4.3	Strategies for overcoming barriers	0.935	
	X4.4	Behaviour changes strategies	0.935	
Commitment (Y1)	Y1.1	Responsibility	0.859	0.788
	Y1.2	Plan of action	0.916	
Independent Wound Care (Y2)	Y2.1	Healthy lifestyle behaviors	0.969	0.931
	Y2.2	Independent wound care behaviors	0.961	

*DFU (Diabetic Foot Ulcer).

In addition, construct reliability was supported by high internal consistency, with Composite Reliability values exceeding the recommended threshold of 0.70 across all constructs, ranging from acceptable to excellent. These results collectively demonstrate that each indicator satisfies the criteria for convergent validity and that the constructs are measured reliably, indicating that the measurement model is suitable for subsequent structural model analyses.

### 3.3 Results of construct reliability testing

Table 3 presents the results of the construct reliability testing using three measures: Cronbach's alpha, Composite Reliability (Rho_a), and Composite Reliability (Rho_c). All constructs demonstrated good reliability, with Cronbach's alpha values exceeding 0.60 and composite reliability values above the recommended threshold of 0.70, indicating adequate to excellent internal consistency. These results confirm that all indicators reliably measured their respective constructs, supporting the robustness of the measurement model.

**Table 3:** Results of construct reliability testing (*n* = 130).

	Cronbach alpha	Composite Reliability (Rho_a)	Composite Reliability (Rho_c)	Interpretation
X1. Individual Factors	0.814	0.826	0.890	Good Reliability
X2. Support and Facilities Factors	0.714	0.734	0.835	Adequate Reliability
X3. Nursing Factors	0.854	0.872	0.902	Very Good Reliability
X4. Supportive-Educative Nursing	0.900	0.918	0.932	Very High Reliability
Y1. Commitment	0.735	0.765	0.881	Good Reliability
Y2. Independent Wound Care	0.926	0.934	0.964	Very High Reliability

### 3.4 Hypothesis testing

Table 4 presents the Results of Structural Model (path analysis) and hypotheses testing. The structural model illustrates the influence of various factors on supportive-educative nursing (X4) and its subsequent impact on independent wound care behaviors (Y2).

**Table 4:** Results of Structural Model (Path Analysis) and Hypothesis Testing (*n* = 130).

Hypothesis testing	*Path Coefficients*	T Statistics (O/SD)	P values	Description
Individual Factors (X1) → Supportive-Educative Nursing (X4)	0.399	3.093	0.002	Significant
Individual Factors (X1) → Independent Wound Care (Y2)	0.376	3.288	0.001	Significant
Support and Facilities Factors (X2) → Supportive-Educative Nursing (X4)	0.227	2.296	0.022	Significant
Support and Facilities Factors (X2)→ Independent Wound Care (Y2)	−0.113	1.160	0.246	Not Significant
Nursing Factors (X3) → Supportive-Educative Nursing (X4)	0.296	3.862	*p* < 0.001	Significant
Nursing Factors (X3) → Independent Wound Care (Y2)	0.088	0.878	0.380	Not Significant
Supportive-Educative Nursing (X4)→ Commitment (Y1)	0.724	17.149	*p* < 0.001	Significant
Commitment (Y1) → Independent Wound Care (Y2)	0.486	4.537	*p* < 0.001	Significant

*T Statistics = original sample (O) ÷ Standard Deviation (SD).

These results answer the following hypothesis:

Hypothesis 1 Individual, support and facilities, and nurse factors are positively associated with supportive–educative nursing. Thus, this hypothesis is supported. Individual factors (X1) significantly predicted supportive-educative nursing (β = 0.399, *t* = 3.093, *p* = 0.002). Support and facilities (X2) and nursing factors (X3) also had significant positive effects, with path coefficients of 0.227 (*p* = 0.022) and 0.296 (*p* < 0.001), respectively. These findings indicate that all three antecedent factors positively contribute to the development of supportive-educative nursing.

Hypothesis 2 Supportive–educative nursing is positively associated with patient commitment. Thus this hypothesis is supported. Supportive-educative nursing (X4) had a strong positive effect on commitment (Y1) (β = 0.724, *t* = 17.149, *p* < 0.001), demonstrating that effective supportive-educative nursing significantly enhances patient commitment.

Hypothesis 3 Patient commitment is positively associated with independent wound care behaviors. Thus this hypothesis is supported. Commitment (Y1) significantly predicted independent wound care (Y2) (β = 0.486, *t* = 4.537, *p* < 0.001), indicating that higher levels of commitment were associated with better self-care wound management.

Hypothesis 4 Supportive–educative nursing and patient commitment mediate the relationships between individual factors, support and facilities factors, nurse factors, and independent wound care behaviors. This hypothesis is partially supported. Individual factors (X1) had a significant direct effect on independent wound care (β = 0.376, *p* = 0.001), whereas support and facilities (X2) and nursing (X3) factors did not (*p* > 0.05). However, through the mediation pathway, supportive-educative nursing (X4) strongly influenced commitment (Y1), which, in turn, significantly affected independent wound care (Y2). These results suggest that supportive-educative nursing and commitment act as mediators, particularly for support and facilities and nursing factors, highlighting their indirect influence on wound care outcomes.

The findings confirm the central role of supportive-educative nursing and patient commitment as mechanisms through which individual, support and facilities, and nursing factors ultimately impact independent wound care behaviors, emphasizing the importance of these constructs in promoting effective nursing outcomes.

### 3.5 The final model of the supportive-educative nursing model based on health promotion

The path analysis showed that Individual Factors (X1) had a significant effect on Supportive-Educative Nursing (X4) with a path coefficient of 0.406 (*t* = 3.154) and also directly influenced Independent Wound Care (Y2) with a coefficient of 0.377 (*t* = 3.646), indicating that individuals with high self-efficacy, prior wound care experience, and strong motivation support educational nursing practices while directly enhancing patient autonomy.

Support and Facilities Factors (X2) significantly affected Supportive-Educative Nursing (X4) with a coefficient of 0.224 (*t* = 2.240) but did not have a direct path to Y2, showing an indirect influence through educational nursing, where adequate facilities, service accessibility, and social support facilitate patient independence.

Nursing Factors (X3) also significantly affected X4 (β = 0.291, *t* = 3.791) without a direct effect on Y2, reflecting the crucial role of nurse guidance, support, and motivation in strengthening educational nursing practices.

Supportive-Educative Nursing (X4) strongly influenced commitment (Y1) with a coefficient of 0.724 (*t* = 17.147), indicating that effective educational nursing substantially enhanced patients’ commitment to self-care. Commitment (Y1) significantly affected Independent Wound Care (Y2) with a coefficient of 0.465 (*t* = 5.186), highlighting that higher commitment promotes greater patient autonomy. Independent Wound Care (Y2), measured by indicators of healthy living and self-care, was explained by 56.2 % of the variables in the model (R² = 0.562).

**Fig. 2:**
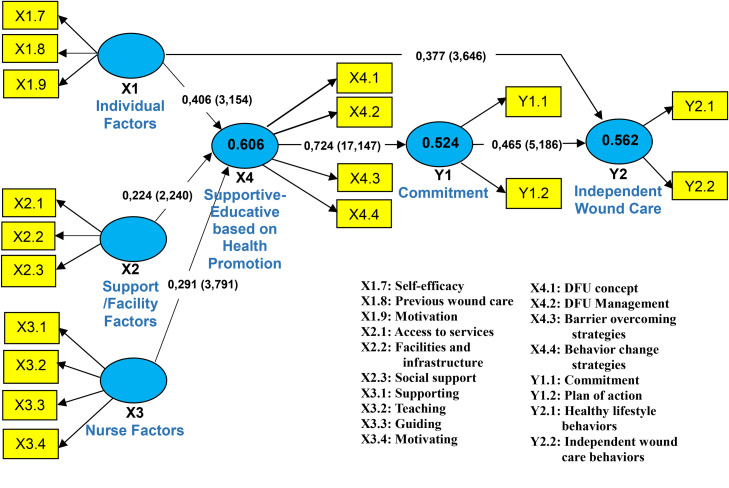
The final construct of the development of a supportive-educative nursing model based on health promotion for independent wound care in diabetic foot ulcer patients.

Overall, the mediation pathways indicate that individual, support and facilities, and nursing factors influence patient independence indirectly through Supportive-Educative Nursing and Commitment, with only Individual Factors exerting a significant direct effect on Y2. This model identifies the four exogenous variables: Individual Factors (X1), Support and Facilities Factors (X2), and Nursing Factors (X3)—as direct predictors of Supportive-Educative Nursing (X4), which in turn affects commitment (Y1), and ultimately commitment directly and significantly influences Independent Wound Care (Y2). After removing the insignificant paths, the final structural model presented in Fig. 2 was considered valid and suitable for further application in nursing practice.

## 4 Discussions

This study developed a supportive–educative nursing model based on health promotion, demonstrating that patient commitment is the key mechanism through which individual, support and facilities, and nurse factors ultimately enhance independent wound care behaviors among patients with diabetic foot ulcers. Rather than acting as a direct determinant, these exogenous factors exert their influence primarily through supportive–educative nursing and the subsequent strengthening of patient commitment. This finding underscores the central role of behavioral mechanisms in sustaining self-care practices beyond clinical encounters.

### 4.1 Interpretation of key findings

The findings indicate that supportive–educative nursing functions as a pivotal enabling process, translating individual readiness, contextual resources, and professional nursing input into meaningful behavioral engagement. This suggests that merely possessing adequate facilities or receiving routine nursing care is insufficient to ensure independence in wound care. Instead, patients require structured education, guidance, and motivational support to actively fosters learning and confidence. Recent studies have similarly emphasized that nursing interventions emphasizing empowerment and participatory education are more effective in improving chronic wound outcomes than task-oriented care alone (Zhuang et al., 2024; Kaewjaladvilai, Chaiviboontham and Sumdaengrit, 2025).

The significant role of patient commitment highlights why improvements in knowledge or skills do not automatically translate into sustained wound-care behaviors. Commitment represents a patient’s internalization of responsibility and intention to act, bridging the gap between nursing input and daily self-care practices. This finding aligns with recent health promotion research showing that commitment to action is a stronger predictor of sustained self-management than is knowledge acquisition alone (Vakilian et al., 2021; Nitichai, Harnirattisai and Hain, 2025).

Notably, nurse-related factors were found to influence independent wound care indirectly rather than directly. This suggests that the effectiveness of nursing support depends less on the presence of nurses per se, and more on how nursing interactions are structured (Nayeri et al., 2020; Izumi, Onishi and Lavery, 2024). Clear communication, consistent guidance, and motivational reinforcement appear to be critical in shaping patients’ engagement in supportive–educative nursing processes. Similar conclusions have been reported in recent studies, indicating that the relational and educational dimensions of nursing care are central to patient self-management outcomes (Osuchukwu et al., 2021; Kusumaningrum, Niken and Paramitta, 2024).

### 4.2 Theoretical integration

The findings of this study are well explained by the integration of Orem’s Self-Care Deficit Nursing Theory and Pender’s health-promotion model. From Orem’s perspective, supportive–educative nursing strengthens patients’ self-care agency by addressing their knowledge deficits, skill limitations, and confidence barriers. Nurses act as facilitators who enable patients to meet their self-care demands, rather than performing care on their behalf.

Pender’s model complements this view by explaining how enhanced self-care agency is transformed into sustained behavior through patient commitment. Commitment to action represents the motivational and cognitive pathways that links nursing support to consistent wound-care practices (Vakilian et al., 2021). Recent empirical work supports this integration, demonstrating that health-promotion-oriented nursing interventions are most effective when they explicitly target both self-care capacity and behavioral commitment, as depicted in Fig. 1 (Zhuang et al., 2024; Kaewjaladvilai, Chaiviboontham and Sumdaengrit, 2025).

By empirically validating this theoretical integration, the present study extends the existing nursing theory by demonstrating how supportive–educative nursing and health promotion jointly contribute to independent wound care in patients with diabetic foot ulcers.

### 4.3 Implications for practice and policy

#### 4.3.1 Implications for practice

The findings suggest that nursing practice should shift from a predominantly task-oriented approach to a structured supportive–educative nursing model that explicitly targets patient commitment. Nurses should be equipped with competencies in patient education, motivational communication, and behavioral support to foster sustained self-care. Incorporating commitment-enhancing strategies such as goal setting, action planning, and reinforcement into routine wound care education may improve long-term outcomes.

#### 4.3.2 Implications for policy and health systems

At the health system level, policies should support the integration of supportive–educative nursing into chronic wound management programs, particularly in the community and primary care settings. Investment in nurse training and standardized educational modules may reduce long-term dependence on clinical services and lower the burden of diabetic foot complications. Recent health system studies have highlighted that empowerment-based care models can improve efficiency and sustainability in chronic disease management, especially in low-income and middle-income countries (Joeliantina et al., 2024).

### 4.4 Strengths and limitations

This study provides an empirically tested theory-driven nursing model that integrates individual, contextual, and professional factors within a coherent framework to support independent wound care in patients with foot ulcers. The integration of nursing and health promotion theories represents a key strength and offers a solid foundation for developing supportive–educative nursing interventions.

However, this study had several limitations. The cross sectional design limits causal inference, and the temporal sequence implied by the model, such as patient commitment leading to independent wound care behaviors, cannot be definitively established with reverse causality remaining a possibility. For example, patients with higher levels of wound care independence may report a stronger commitment rather than commitment preceding independence. In addition, reliance on self-reported measures, including wound care independence, may be subject to a social desirability bias. Future longitudinal or interventional studies incorporating objective outcome measures are required to confirm the directionality and robustness of the proposed relationships.

## 5 Conclusion

This study developed and empirically tested a health promotion–based supportive–educative nursing model for independent wound care in patients with diabetic foot ulcers. The findings demonstrated significant associations among individual factors, support and facilities factors, nurse factors, supportive–educative nursing, patient commitment, and independent wound care behaviors. In particular, patient commitment emerged as a central construct within the model, linking supportive–educative nursing with patients’ engagement in independent wound care.

Given the cross-sectional design, the findings should be interpreted as associative rather than causal, and no direct effects or improvements should be inferred. Nonetheless, the model provides a theoretically grounded and empirically supported framework that clarifies how nursing support and health promotion mechanisms are related to independent wound-care behaviors.

The proposed model offers a valuable foundation for future longitudinal and interventional studies aimed at testing causal pathways and evaluating the effectiveness of supportive–educative nursing interventions. From a practical perspective, the model highlights the importance of structured supportive–educative nursing and patient commitment as key components to considered when designing strategies to support independent wound care in patients with diabetic foot ulcers.

## Declaration of generative AI and AI-assisted technologies in the writing process

During the development of this manuscript, (s) utilized DeepSeek to enhance the clarity and readability of text. The author(s) subsequently reviewed and revised all the content as necessary and assumed full responsibility for the final version of the manuscript.

## Funding

This research did not receive any specific grant from funding agencies in the public, commercial, or not-for-profit sectors.

## CRediT authorship contribution statement

**Novita Verayanti Manalu:** Writing – original draft, Visualization, Resources, Project administration, Methodology, Investigation, Funding acquisition, Formal analysis, Data curation, Conceptualization. **Esti Yunitasari:** Writing – review & editing, Supervision. **Sriyono Sriyono:** Writing – review & editing, Validation, Supervision. **Nursalam Nursalam:** Validation. **Ferry Efendi:** Validation. **Yunus Elon:** Writing – original draft, Resources, Methodology, Investigation, Formal analysis, Data curation.

## Declaration of competing interest

The authors declare that they have no known competing financial interests or personal relationships that could have appeared to influence the work reported in this manuscript.

## Data Availability

Data supporting the results of this study can be obtained from the corresponding author upon reasonable request.

## Appendix Supplementary materials

Image, application 1

Image, application 2

Image, application 3

Image, application 4
