# Supplementary material for: Development of a supportive-educative nursing model based on health promotion for independent wound care in diabetic foot ulcer patients: A cross-sectional study
Source: Int J Nurs Stud Adv. 2026 Feb 9;10:100504. doi: 10.1016/j.ijnsa.2026.100504 (PMC12936470; doi:10.1016/j.ijnsa.2026.100504)
Supplement: Supplementary file 1 [file mmc1.pdf]

PEMERINTAH KOTA BANDAR LAMPUNG
DINAS KESEHATAN
Jalan Way Pengubuan No. 3 Pahoman, Enggal Bandar Lampung, 35127
Telepon (0721) 472003, Website : www.dinkes.bandarlampungkota.go.id

Bandar Lampung, 26 Februari 2025

Nomor : B/ /400.7.22/III.02.V/02/2025
Sifat : Biasa
Lampiran : -
Hal : Izin Penelitian

Yth. Wakil Dekan I Universitas Airlangga
di
Surabaya

Sehubungan dengan surat saudara nomor : 999/B/UN3.FKp/I/TA.00.03/2025 tanggal 14 Februari 2025 perihal pengambilan data penelitian dalam rangka penelitian bagi mahasiswa Program Studi Doktor Keperawatan Fakultas Keperawatan Universitas Airlangga, atas nama **NOVITA VERAYANTI MANALU** NIM. 332221007 Judul Penelitian : *"Pengembangan Model Supportive Educative Berbasis Planned Behavior terhadap Kemandirian Perawatan Luka Penderita Diabetic Foot Ulcer".*

Perlu kami Informasikan beberapa hal sebagai berikut :

a. Izin Pengambilan data di Wilayah Dinas Kesehatan Kota Bandar Lampung mengacu kepada Peraturan Dinas Kesehatan Kota Bandar Lampung.
b. Pengambilan data digunakan semata-mata hanya untuk kepentingan Akademik/Studi dan tidak akan dipublikasikan tanpa izin tertulis dari Kepala Dinas Kesehatan Kota Bandar Lampung.
c. Kegiatan Pengambilan data dilaksanakan selama 3 (tiga) bulan sejak tanggal ditetapkan.
d. Setelah menyelesaikan kegiatan tersebut, mahasiswa diwajibkan menyampaikan laporan hasil kegiatannya kepada Kepala Dinas Kesehatan Kota Bandar Lampung.

Demikian atas perhatian dan kerjasamanya diucapkan terima kasih.

an. Kepala Dinas Kesehatan
Sekretaris,

drg. Santi Sundari., M.Kes
Pembina TK.I (IV/b)
NIP. 19790614 200604 2 010

Tembusan : disampaikan kepada Yth,
1. Sdr. Kabid. Pelayanan Kesehatan
2. Sdr. Kabid. Kesehatan Masyarakat
3. Sdr. Kabid. Pencegahan dan Pengendalian Penyakit
4. Sdr. Kabid. Sumber Daya Kesehatan
5. Sdr. Kepala Puskesmas Se-Kota Bandar Lampung
6. Sdr. Dosen Pembimbing
7. Sdr. Mahasiswa Yang bersangkutan
8. ------ Pertinggalan ----------

Dipindai dengan CamScanner
