# Supplementary material for: Development of a supportive-educative nursing model based on health promotion for independent wound care in diabetic foot ulcer patients: A cross-sectional study
Source: Int J Nurs Stud Adv. 2026 Feb 9;10:100504. doi: 10.1016/j.ijnsa.2026.100504 (PMC12936470; doi:10.1016/j.ijnsa.2026.100504)
Supplement: Supplementary file 2 [file mmc2.pdf]

PEMERINTAH KOTA BANDARLAMPUNG
DINAS PENANAMAN MODAL DAN PELAYANAN TERPADU SATU PINTU
Jalan Dr. Susilo Nomor 2 Bandarlampung, Telepon (0721) 476362
Faksimile (0721) 476362 Website: www.dpmptsp.bandarlampungkota.go.id
Pos-el: dpmptsp.kota@bandarlampungkota.go.id

SURAT KETERANGAN PENELITIAN (SKP)
Nomor :1871/070/03953/SKP/III.16/VI/2025

Berdasarkan Peraturan Menteri Dalam Negeri Republik Indonesia Nomor 03 Tahun 2018 tentang Penerbitan Surat Keterangan Penelitian dan Rekomendasi dari Kepala Badan Kesatuan Bangsa Dan Politik Kota Bandar Lampung Nomor 070/00465/IV.05/2025 Tanggal 2025-02-17 14:00:28, yang bertandatangan dibawah ini Kepala Dinas Penanaman Modal dan Pelayanan Terpadu Satu Pintu Kota Bandar Lampung memberikan Surat Keterangan Penelitian (SKP) kepada :

1.	Nama	: NOVITA VERAYANTI MANALU
2.	Alamat	: JL PURI WISATA BLOK II NO 04 RT 18 KEL./DESA WAY HALIM PERMAI KEC. WAY HALIM KAB/KOTA KOTA BANDAR LAMPUNG PROV. LAMPUNG
3.	Judul Penelitian	: PENGEMBANGAN MODEL SUPORTIVE EDUKATIVE BERBASIS HEALTH PROMOTION TERHADAP KEMANDIRIAN PERAWATAN LUKA PENDERITA DIABETIK FOOT ULCER
4.	Tujuan Penelitian	: PENYEBARAN KUESIONER DAN PENELITIAN PENDERITA DIABETES DAN ULKUS KAKI DIABETES (DFU) DI PROVINSI LAMPUNG DAN DINAS KESEHATAN KOTA BANDAR LAMPUNG
5.	Lokasi Penelitian	: PADA DINAS KESEHATAN PROVINSI LAMPUNG , DINAS KESEHATAN KOTA BANDAR LAMPUNG DAN PUSKESMAS DI KOTA BANDAR LAMPUNG
6.	Tanggal dan/atau lamanya penelitian	: 1 (satu) TAHUN
7.	Bidang Penelitian	: KEPERAWATAN
8.	Status Penelitian	: -
9.	Nama Penanggung Jawab atau Koordinator	: Dr. IKA YUNI W., S.Kep.Ns.,M.Kep.,Sp.Kep.MB
10.	Anggota Penelitian	: NOVITA VERAYANTI MANALU
11.	Nama Badan Hukum, Lembaga dan Organisasi Kemasyarakataan	: UNIVERSITAS AIRLANGGA

Dengan Ketentuan sebagai berikut :
1. Pelaksanaan Penelitian tidak disalahgunakan untuk tujuan tertentu yang dapat mengganggu stabilitas pemerintah.
2. Setelah Penelitian selesai, agar menyerahkan hasilnya kepada Badan Kesatuan Bangsa Dan Politik (BAKESBANGPOL) Kota Bandar Lampung.
3. Surat Keterangan Penelitian ini berlaku selama 1 (satu) tahun sejak tanggal ditetapkan.

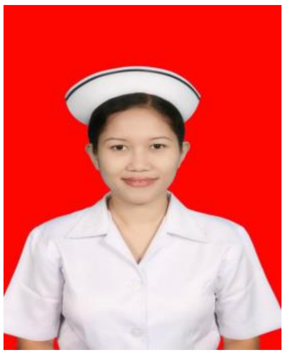

Ditetapkan di : Bandarlampung
pada tanggal : 17 Februari 2025

Ditandatangani secara elektronik oleh :
Kepala Dinas

MUHTADI A. TEMENGGUNG, S.T., M.Si.
NIP 19710810 199502 1 001

Tembusan :
1. BAKESBANGPOL Kota Bandar Lampung
2. Bapeda Kota Bandar Lampung
3. Pertinggal
