# Supplementary material for: Development of a supportive-educative nursing model based on health promotion for independent wound care in diabetic foot ulcer patients: A cross-sectional study
Source: Int J Nurs Stud Adv. 2026 Feb 9;10:100504. doi: 10.1016/j.ijnsa.2026.100504 (PMC12936470; doi:10.1016/j.ijnsa.2026.100504)
Supplement: Supplementary file 3 [file mmc3.pdf]

KOMISI ETIK PENELITIAN KESEHATAN
HEALTH RESEARCH ETHICS COMMITTEE

FAKULTAS KEPERAWATAN UNIVERSITAS AIRLANGGA
FACULTY OF NURSING UNIVERSITAS AIRLANGGA

KETERANGAN LOLOS KAJI ETIK
DESCRIPTION OF ETHICAL APPROVAL

"*ETHICAL APPROVAL*"
Nomor: 3664-KEPK

Komite Etik Penelitian Kesehatan Fakultas Keperawatan Universitas Airlangga dalam upaya melindungi hak asasi dan kesejahteraan subyek penelitian kesehatan, telah mengkaji dengan teliti protokol berjudul:

The Committee of Ethical Approval in the Faculty of Nursing Universitas Airlangga, with regards of the protection of Human Rights and welfare in health research, carefully reviewed the research protocol entitled:

"PENGEMBANGAN MODEL KEPERAWATAN SUPORTIF EDUKATIF BERBASIS PROMOSI KESEHATAN TERHADAP KEMANDIRIAN PERAWATAN LUKA PASIEN ULKUS KAKI DIABETES"

Peneliti utama
Principal Investigator : **Novita Verayanti Manalu, S.Kp., Ns., MAN**

Nama Institusi
Name of the Institution : Fakultas Keperawatan, Universitas Airlangga

Unit/Lembaga/Tempat Penelitian
Setting of research : Puskesmas Wilayah Kota Bandar Lampung

Dan telah menyetujui protokol tersebut di atas melalui Dipercepat
And approved the above-mentioned protocol with Expedited

Surabaya, 10 Februari 2025
Ketua, (*CHAIRMAN*)

UNIVERSITAS AIRLANGGA
KOMISI ETIK PENELITIAN KESEHATAN
FAKULTAS KEPERAWATAN

Nuzul Qur'aniati, S.Kep.Ns.,M.Ng.,PhD
NIP. 1978 0208 2014 09 2001

*** Masa berlaku 1 tahun**
1 year validity period
