# Supplementary material for: Development of a supportive-educative nursing model based on health promotion for independent wound care in diabetic foot ulcer patients: A cross-sectional study
Source: Int J Nurs Stud Adv. 2026 Feb 9;10:100504. doi: 10.1016/j.ijnsa.2026.100504 (PMC12936470; doi:10.1016/j.ijnsa.2026.100504)
Supplement: Supplementary file 4 [file mmc4.docx]

# Table S1. Description of Questionnaire Sources, Adaptations, and Item Composition

| **Construct** | **Sub-constructs** | **Indicators** | **Original Source** | **Adaptation / Modification** | **Question Number** | **Rated** | **Measurement Focus** |
| --- | --- | --- | --- | --- | --- | --- | --- |
| **Individual Factors**  **(X1)** | 1. Self-efficacy 2. Previous wound care experience 3. Motivation | 1. Dietary habit 2. Physical activities 3. Blood sugar check 4. Treatment program 5. Smoking 6. Habit nurse wound 7. Use tool maintenance wound 8. Belief 9. Encouragement self for guard pattern Eat 10. Encouragement self for do activities 11. Encouragement self for blood sugar check 12. Encouragement self for treatment program 13. Encouragement self for nurse wound Alone 14. Self-encouragement | DMSES; SDSCA  VA-DFCS; TSRQ  DIAB-Q | Adapted to diabetic foot ulcer wound care context and self-management behaviors | 1-6  7-9  10-12  13-15  16  1-17  18-23  1-3  4-6  7-9  10-12  13-15  16-18 | 5-point Likert scale (1=never to 5=always)  5-point Likert scale (1=never to 5=always)  5-point Likert scale (1=strongly disagree to 5=strongly agree) | Confidence, experience, and motivation to perform wound care |
| **Support and Facilities Factors**  **(X2)** | 1. Access to services 2. Facilities and infrastructure 3. Social support | 1. Access to facility health 2. Access to services and information 3. Supporting the facilities' healthy food 4. Supporting facilities' physical activities 5. Supporting facilities' stress management 6. Emotional 7. Instrumental 8. Informational 9. Evaluation and reward | PAHCQ; Policy-based instruments  DSSQ | Contextualized to local health service access and family support | 1-4  5-8  1-2  3-5  6-7  1-7  8-13  14-17  18-23 | 2-point Binary scale (1=Yes and 0=No)  2-point Binary scale (1=Yes and 0=No)  5-point Likert scale (1=never to 5=always) | Environmental and social resources supporting wound care |
| **Nurse Factors**  **(X3)** | 1. Support 2. Teaching 3. Guiding 4. Motivating | 1. Support emotional 2. Support information 3. Method 4. Understanding 5. Material 6. Instructions 7. Push maintenance independent 8. Reduce complications | PPIS; NADFPQ;  PACIC  IDF-based tools  IDF-based tools  ADDQoL | Modified to assess nurse facilitation of DFU self-care | 1-4  5-8  1-4  5-7  1-7  8-11  1-4  5-10 | 5-point Likert scale (1=never to 5=always)  5-point Likert scale (1=never to 5=always)  5-point Likert scale (1=never to 5=always)  5-point Likert scale (1=never to 5=always) | Professional nursing support and education |
| **Supportive–Educative Nursing**  **(X4)** | 1. DFU concept 2. DFU management 3. Coping strategies 4. Behavior change | 1. Definition of DFU 2. Causes of DFU 3. Signs & Symptoms 4. Complications 5. Supporting (Cognition & Affection) (Diet, physical activity, blood sugar check, treatment program, care wound independent) 6. Teaching (Interpersonal) (Diet, physical activity, blood sugar check, treatment program, care wound independent) 7. Guiding (Situational) (Diet, physical activity, blood sugar check, treatment program, care wound independent) 8. Supporting (Cognition & Affection) 9. Teaching (Interpersonal) 10. Guiding (Situational) 11. Precontemplation 12. Contemplation 13. Preparation for Action 14. Action 15. Maintenance | DFSQ-UMA; DiaFootQ; PAID; HAPA-related tools;  HBSCQ; BCSHES  DIBQ | Integrated Orem’s SCDNT and Pender’s HPM concepts | 1-4  5-10  11-13  14-15  1-11  2-18  19-23  1-3  4-9  10-13  1-2  3-4  5-6  7-8  9-10 | 5-point Ordinal scale (1=poor to 5=excellent)  2-point Binary scale (1=Yes and 0=No)  2-point Binary scale (1=Yes and 0=No)  2-point Binary scale (1=Yes and 0=No) | Supportive–educative processes enabling self-care |
| **Patient Commitment (Y1)** | 1. Responsibility 2. Plan of action | 1. Management pattern life Healthy 2. Management maintenance wound independent 3. Look for help 4. Arranging steps 5. Make timetable 6. Emergency measures emergency 7. Belief | DFSQ-UMA; HCQ-HRD  HAPA; PAID; HBSCQ | Modified to reflect commitment in DFU wound care | 1-3  4  5  1-2  3  4  5 | 2-point Binary scale (1=Yes and 0=No)  2-point Binary scale (1=Yes and 0=No) | Behavioral commitment to independent wound care |
| **Independent Wound Care (Y2)** | 1. Healthy lifestyle behavior 2. Self-wound care | 1. Eating habit 2. Physical activities 3. Blood sugar check 4. Treatment program 5. Assessment wound 6. Wash wound 7. Use topical wound medication 8. Bandaging wound 9. Control infection 10. Off-loading / reducing foot pressure | HPLP-II;  DIBQ  DFSBS; DiaFootQ | Adapted to DFU stages and wound care activities | 1-4  5-6  7-8  9-11  1-3  4-6  7-8  9-11  12-13  14-15 | 5-point Likert scale (1=never to 5=always)  5-point Likert scale (1=never to 5=always) | Sustained independent wound care behaviors |

***Questionnaire and Instrument Abbreviations**

ADDQoL - The Audit of Diabetes Dependent Quality of Life (Setyawati et al., 2024)

BCSHES - Brief Coping Strategies in Health-Related Events Scale (Huijg et al., 2014)

DFSBS - Diabetic Foot Self-Behavior Scale (Lecker et al., 2022)

DFSQ-UMA - Diabetic Foot Self-Care Questionnaire (Jiang et al., 2019)

DIAB-Q - Diabetes Questionnaire (Steinberg et al., 2010)

DiaFootQ - Diabetic Foot Questionnaire (Ruiz-Mun ̃oz et al., 2024)

DIBQ - Determinants of Implementation Behavior Questionnaire

DMSES - Diabetes Management Self-Efficacy Scale (van der Bijl et al., 1999)

DSSQ - Diabetes Social Support Questionnaire (La Greca & Bearman, 2002)

HAPA - Health Action Process Approach (Schwarzer, 2016)

HCQ-HRD Health Care Questionnaire for High-Risk Diabetic Feet (Mahmoodi et al., 2021)

HBSCQ - Health Behavior and Stages of Change Questionnaire (Royal & Royal, 2017)

HPLP-II - Health-Promoting Lifestyle Profile II (Walker et al., 1988)

IDF - International Diabetes Federation (Bigyananda Meitei, 2019)

NADFPQ - Nurse Assessment of Diabetic Foot Prevention Questionnaire (Hidalgo-Ruiz et al., 2023)

PACIC - Patient Assessment of Chronic Illness Care (Glasgow et al., 2005)

PAHCQ - Patient Assessment of Health Care for Chronic Conditions Questionnaire (Hoseini-Esfidarjani et al., 2021)

PAID - Problem Areas in Diabetes Scale (Polonsky et al., 1995)

PPIS - Patient–Provider Interaction Scale (Bakken et al., 2000)

SDSCA - Summary of Diabetes Self-Care Activities (Toobert et al., 2000)

TSRQ - Treatment Self-Regulation Questionnaire (Ryan & Connell, 1989)

VA-DFCS - VA-Diabetes Foot Care Survey (Chin et al., 2019)

***Conceptual and Theoretical Abbreviation**

DFU - Diabetic Foot Ulcer

HPM - Health Promotion Model (Pender)

SCDNT - Self-Care Deficit Nursing Theory (Orem)

**Reference**

Bakken, S., Holzemer, W. L., Brown, M.-A., Powell-Cope, G. M., Turner, J. G., Inouye, J., Nokes, K. M., & Corless, I. B. (2000). Relationships Between Perception of Engagement with Health Care Provider and Demographic Characteristics, Health Status, and Adherence to Therapeutic Regimen in Persons with HIV/AIDS. *AIDS Patient Care and STDs*, *14*(4), 189–197. https://doi.org/https://doi.org/10.1089/108729100317795

Bigyananda Meitei, W. (2019). *District Level Assessment of Spatial Clustering and Determinants of Diabetes Mellitus among Older Adolescents and Young Adults in India*. *1*(Idf 2015), 15–25. https://doi.org/10.32789/publichealth.2019.1003

Chin, Y.-F., Huang, T.-T., Hsu, B. R.-S., Weng, L.-C., & Wang, C.-C. (2019). Factors associated with foot ulcer self-management behaviours among hospitalised patients with diabetes. *Journal of Clinical Nursing*, *28*(11–12), 2253–2264. https://doi.org/10.1111/jocn.14822

Glasgow, R. E., Wagner, E. H., Schaefer, J., Mahoney, L. D., Reid, R. J., & Greene, S. M. (2005). Development and Validation of the Patient Assessment of Chronic Illness Care (PACIC). *Medical Care*, *43*(5), 436–444. https://doi.org/10.1097/01.mlr.0000160375.47920.8c

Hidalgo-Ruiz, S., Ramírez-Durán, M. d. V., Basilio-Fernández, B., Alfageme-García, P., Fabregat-Fernández, J., Jiménez-Cano, V. M., Clavijo-Chamorro, M. Z. ., & Gomez-Luque, A. (2023). Assessment of Diabetic Foot Prevention by Nurses. *Nurs. Reports*, *13*, 73–84. https://doi.org/doi.org/10.3390/nursrep13010008

Hoseini-Esfidarjani, S.-S., Negarandeh, R., Delavar, F., & Janani, L. (2021). Psychometric evaluation of the perceived access to health care questionnaire. *BMC Health Services Research*, *21*(1), 638. https://doi.org/https://doi.org/10.1186/s12913-021-06655-2

Huijg, J. M., Gebhardt, W. A., Dusseldorp, E., Verheijden, M. W., Zouwe, N. van der, Middelkoop, B. J., & Crone, M. R. (2014). Measuring determinants of implementation behavior: psychometric properties of a questionnaire based on the theoretical domains framework. *Implementation Science*, *9*(33), 1–15. https://doi.org/10.1186/1748-5908-9-33

Jiang, X., Wang, J., Lu, Y., Jiang, H., & Li, M. (2019). Self-efficacy-focused education in persons with diabetes: A systematic review and meta-analysis. *Psychology Research and Behavior Management*, *12*, 67–79. https://doi.org/https://doi.org/10.2147/PRBM.S192571

La Greca, A. M., & Bearman, K. J. (2002). The Diabetes Social Support Questionnaire–Family Version: Evaluating adolescents’ diabetes‐specific support from family members. *Journal of Pediatric Psychology*, *27*(8), 665–676. https://doi.org/https://doi.org/10.1093/jpepsy/27.8.665

Lecker, L., Stevens, M., Thienel, F., Lazovic, D., AkkerScheek, I. van den, & SeeberI, G. H. (2022). Validity and reliability of the German translation of the Diabetes Foot Self-Care Behavior Scale (DFSBS-D). *PLoS ONE*, *17*(6), 1–14. https://doi.org/https://doi.org/10.1371/journal.pone.0269395 J

Mahmoodi, H., Abdi, K., Navarro-Flores, E., Karimi, Z., Nia, H. S., & Gheshlagh, R. G. (2021). Psychometric evaluation of the Persian version of the diabetic foot self-care questionnaire in Iranian patients with diabetes. *BMC Endocrine Disorders*, *21*(72), 1–7. https://doi.org/https://doi.org/10.1186/s12902-021-00734-5

Polonsky, W. H., Anderson, B. J., Lohrer, P. A., Welch, G., Jacobson, A. M., Aponte, J. E., & Schwartz, C. E. (1995). Assessment of Diabetes-Related Distress. *Diabetes Care*, *18*(6), 754–760. https://doi.org/https://doi.org/10.2337/diacare.18.6.754

Royal, K. D., & Royal, R. A. (2017). An Evaluation of the Psychometric Properties of the Behavior Change Strategies for Healthy Eating Scale. *Journal of Nursing Measurement*, *3*, 411–420. https://doi.org/10.1891/1061-3749.25.3.411

Ruiz-Mun ̃oz, M., Fernandez-Torres, R., Formosa, C., Gatt, A., Alberto, Perez-Paneroa, J. ́e ́, Perez-Bellosoc, A. J., Martínez-Barriosa, F. J., & Gonza ́lez-Sanchez, M. (2024). Development and validation of a new questionnaire for the assessment of patients with diabetic foot disease: The Diabetic Foot Questionnaire (DiaFootQ). *Primary Care Diabetes*, *18*, 525–532. https://doi.org/https://doi.org/10.1016/j.pcd.2024.07.002

Ryan, R. M., & Connell, J. P. (1989). Perceived locus of causality and internalization: Examining reasons for acting in two domains. *Journal of Personality and Social Psychology*, *57*(5), 749–761. https://doi.org/https://doi.org/10.1037/0022-3514.57.5.749

Schwarzer, R. (2016). The Health Action Process Approach (HAPA): A social-cognitive approach to health behavior change. *Actualidades En Psicología*, *30*(121), 119–130. https://doi.org/http://dx.doi.org/10.15517/ap.v30i121.23458

Setyawati, A., Saleh, A., Tahir, T., Yusuf, S., Syahrul, S., Aminuddin, A., Raihan, M., Jafar, N., Hamzah, H., & Arfian, N. (2024). Matrix Metalloproteinase-9 Testing of Golden Rice Cookies With Piper Crocatum Active Extract for Preventing Foot Ulcers in Patients With Diabetes: Protocol for a Randomized Controlled Trial. *JMIR Research Protocols*, *13*. https://doi.org/10.2196/49940

Steinberg, J., Edmonds, M., Hurley, D. P., & King, W. N. (2010). *Confirmatory data from EU study supports Apligraf for the treatment of neuropathic diabetic foot ulcers.* https://doi.org/10.7547/1000073

Toobert, D. J., Hampson, S. E., & Glasgow, R. E. (2000). The summary of diabetes self-care activities measure: Results from 7 studies and a revised scale. *Diabetes Care*, *23*(7), 943–950. https://doi.org/10.2337/diacare.23.7.943

van der Bijl, J., van Poelgeest-Eeltink, A., & Shortridge-Baggett, L. (1999). The psychometric properties of the diabetes management self-ef®cacy scale for patients with type 2 diabetes mellitus. *Journal of Advanced Nursing*, *30*(2), 352–259. https://doi.org/https://doi.org/10.1046/j.1365-2648.1999.01077.x

Walker, S. N., Volkan, K., Sechrist, K. R., & Pender, N. J. (1988). Health-promoting life styles of older adults: comparisons with young and middle-aged adults, correlates and patterns. *NS Adv Nurs Sci*, *11*(1), 79–90. https://doi.org/10.1097/00012272-198810000-00008
